# Protocol for capturing the RNA-binding proteome from plants using orthogonal organic phase separation

**DOI:** 10.1016/j.xpro.2025.104231

**Published:** 2025-11-27

**Authors:** Victor A. Sánchez-Camargo, Gertjan Kramer, Harrold A. van den Burg

**Affiliations:** 1 Molecular Plant Pathology, Swammerdam Institute for Life Sciences, University of Amsterdam, 1098 XH Amsterdam, the Netherlands; 2 Laboratory for Mass Spectrometry of Biomolecules, Swammerdam Institute for Life Sciences, University of Amsterdam, 1098 XH Amsterdam, the Netherlands; 3 Rijk Zwaan Breeding B.V., Crezéelaan 40, 2678 KX De Lier, the Netherlands

**Keywords:** Plant sciences, Molecular biology, Protein biochemistry

## Abstract

RNA-binding proteins (RBPs) regulate many processes related to RNA biogenesis, function, localization, and degradation. Here, we present a protocol for the *en masse* isolation of RBPs crosslinked to RNA and provide a strategy for subsequent proteomics analysis. We describe the steps for *in vivo* UV crosslinking of RNA-protein complexes, tissue lysis, fractionation, and purification of crosslinked RNA-protein adducts using organic solvents. Although the protocol was developed for *Nicotiana benthamiana* leaves, it can be optimized for use in different plants and tissues.

## Highlights

• Guide for standardization of *in vivo* UV crosslinking of RNA-protein complexes in plants
• Step-by-step protocol for capturing RNA-binding proteins from plant leaves
• Purification of high-quality RNA, proteins, and RNA-binding proteins
• Guidelines for RNA-binding protein enrichment analysis

## Abstract (editor highlights)

Publisher’s note: Undertaking any experimental protocol requires adherence to local institutional guidelines for laboratory safety and ethics.

## Graphical abstract

**Figure:**
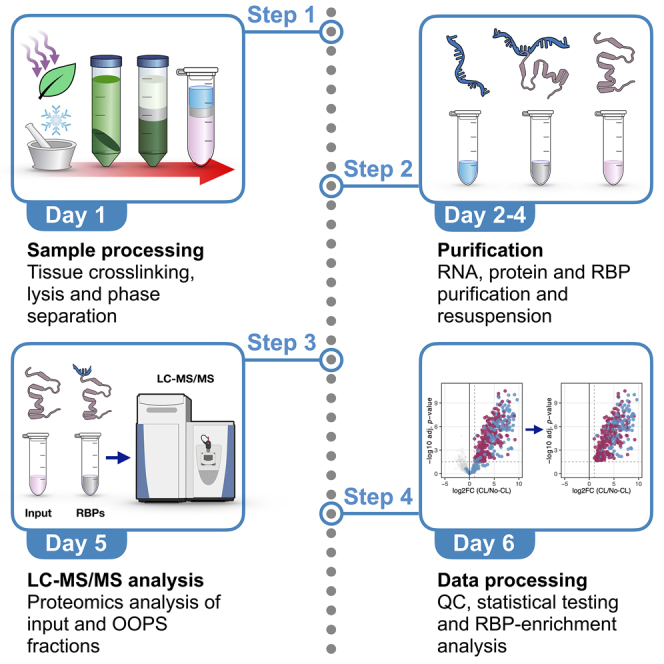

## Before you begin

RNA-binding proteins (RBPs) regulate RNA-related processes such as RNA splicing, nuclear export, stability, relocalization to e.g., processing (P-)bodies, and translation, as well as post-transcriptional gene silencing.^1^ They function by recognizing specific RNA motifs or structures through canonical RNA recognition domains and also through low-complexity regions such as intrinsically disordered domains.^2^^,^^3^^,^^4^ Despite their essential roles in development, environmental adaptation, and stress responses, the functional diversity and molecular mechanisms of plant RBPs remain poorly characterized.^5^^,^^6^^,^^7^^,^^8^

**Figure 1:**
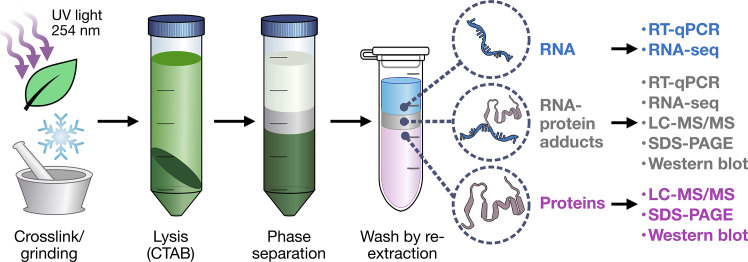
Overview of the OOPS protocol Plant leaves are harvested and crosslinked using UV light at 254 nm. The tissue is then frozen and ground, and lysis is performed using a custom CTAB buffer, followed by clarification of the lysate. Phase separation with phenol-chloroform produces two main phases (aqueous and organic) and an interphase containing the crosslinked RNA-protein fraction. Successive rounds of organic re-extraction remove contaminants from the interphase. High-quality protein and RNA are produced for downstream analysis. RNA and protein drawings were retrieved from NIAID NIH BioArt Source.^12^^,^^13^

Orthogonal Organic Phase Separation (OOPS) is a method that allows the global identification of RBPs without relying on specific protein tags or polyadenylated RNA, which is the case for RNA interactome capture.^9^^,^^10^^,^^11^ It uses UV light to crosslink RNA-protein complexes *in vivo*, followed by repeated phenol-chloroform extractions, during which RNA-protein adducts accumulate at the interphase. This separation enables the analysis of both proteins and RNA present at the interphase (Figure 1). This type of approach and other variants have been successfully used in animal and bacterial systems,^10^^,^^14^ and more recently in plants, where over 2,000 RBPs have been identified from Arabidopsis tissues.^11^^,^^15^^,^^16^^,^^17^

### Innovation

A major challenge in applying RNA-protein capture methods to plant systems is the rigid cell wall, which complicates tissue lysis and limits UV crosslinking efficiency. Additionally, plant tissues are rich in secondary metabolites, such as phenolics, pigments, and polysaccharides, which interfere with protein extraction and downstream proteomic analysis.^18^

Here, we developed a modified, cost-effective OOPS protocol for *Nicotiana benthamiana* incorporating an acidic cetyltrimethylammonium bromide (CTAB)-based lysis step. CTAB, a cationic detergent, removes saccharides and remains compatible with high salt concentrations, enabling protein extraction at high ionic strength.^19^ In addition, polyvinylpolypyrrolidone (PVPP), a synthetic polymer, was included to bind polyphenols and other secondary metabolites that may interfere with downstream analyses.^18^ Combined with elevated temperatures, these conditions enhance the release of organellar content and disrupt non-covalent molecular interactions arising during cell disruption, improving the purity of RNA-protein complexes obtained by OOPS, increasing reproducibility. Compared with other phase separation-based protocols, this workflow provides more stringent extraction conditions, enhancing the recovery of nuclear, organellar, and membrane-associated proteins, at a fraction of the cost (approximately 2 USD versus 100 USD per sample). Moreover, because the procedure is entirely centrifuge-based, it can be readily implemented in standard molecular biology laboratories without compromising throughput.

At the end of this protocol, the user obtains a high-quality preparation of RNA-binding proteins suitable for proteomics and other applications, together with the corresponding RNA fraction, which can be used for RT-qPCR or RNA sequencing. Finally, we provide a demonstration of RBP enrichment analysis with proteomics data obtained from *N. benthamiana*.

### Plant growth

**Timing: 5 weeks**

1. Sow *N. benthamiana* seeds in well-watered potting soil and grow the plants at 25°C under long day photoperiod conditions (16 h light/8 dark).

***Note:*** A typical experiment includes both UV-crosslinked and non-crosslinked samples. Grow 12 plants per treatment and replicate. We recommend a minimum of four biological replicates per condition.

2. After two weeks, transplant the seedlings into individual pots containing the same soil type, and continue growing them for an additional three weeks.

***Note:*** This protocol was developed using the fifth and sixth true leaves (counting from bottom-to-top) of 5-week-old plants.

## Key resources table

**Table:** 

REAGENT or RESOURCE	SOURCE	IDENTIFIER
**Chemicals, peptides, and recombinant proteins**		

2-mercaptoethanol	Sigma-Aldrich	Cat# M6250
3-(N-morpholino) propanesulfonic acid (MOPS)	Millipore	Cat# 475899
Cetyltrimethylammonium bromide (CTAB)	Sigma-Aldrich	Cat# H6269
Chloroform (analytic grade)	Supelco	Cat# 1024451000
Diethyl pyrocarbonate (DEPC)	Sigma-Aldrich	Cat# D5758
Ethanol absolute (analytic grade)	Supelco	Cat# 1.00983
Ethylenediaminetetraacetic acid (EDTA)	Sigma-Aldrich	Cat# E4884
dNTPs	Thermo Fisher Scientific	Cat# R0192
Glacial acetic acid	VWR	Cat# 450013Y
Glycogen, molecular biology grade	Thermo Fisher Scientific	Cat# R0561
Hydrochloric acid (HCl)	VWR	Cat# VWRC20255.290
Isopropanol (analytic grade)	Supelco	Cat# 109634
Liquid nitrogen	Any brand	N/A
Methanol (analytic grade)	Supelco	Cat# 1.06035
Oligo dT	Thermo Fisher Scientific	Cat# 18418012
Phenylmethylsulfonyl fluoride (PMSF)	Sigma-Aldrich	Cat# P7626
Polyvinylpolypyrrolidone (PVPP)	Supelco	Cat# 77627
Random hexamers	Invitrogen	Cat# N8080127
ROTI Aqua-Phenol (or similar water-saturated phenol)	Carl Roth	Cat# A980.3
Sodium citrate	Supelco	Cat# 1.06448
Triethylammonium bicarbonate (TEAB)	Thermo Fisher Scientific	Cat# 18597
Tris(hydroxymethyl)aminomethane (Tris)	Millipore	Cat# 648310-M
Sodium chloride (NaCl)	Supelco	Cat# 1.06404
Sodium dodecyl sulfate (SDS)	Sigma-Aldrich	Cat# L5750
Sodium hydroxyde (NaOH)	Sigma-Aldrich	Cat# 221465
Trizol LS reagent	Thermo Fisher Scientific	Cat# 10296028
UltraPure DNase/RNase-free distilled water	Invitrogen	Cat# 10977015

**Critical commercial assays**		

Complete protease inhibitor cocktail (EDTA-free)	Roche	Cat# 11873580001
DNase I, RNAse-free	Thermo Fisher Scientific	Cat# EN0521
DNase I 10× buffer	Thermo Fisher Scientific	Cat# B43
RNase A/T1 mix	Thermo Fisher Scientific	Cat# EN0551
Proteinase K	Thermo Fisher Scientific	Cat# EO0492
RevertAid First Strand cDNA synthesis kit	Thermo Fisher Scientific	Cat# K1621
RiboLock RNase inhibitor	Thermo Fisher Scientific	Cat# EO0382
HOT FIREPol EvaGreen qPCR supermix	Solis BioDyne	Cat# 08-36-00001

**Experimental models: Organisms/strains**		

*Nicotiana benthamiana* (wild type, LAB)	Bally et al.^20^	N/A

**Oligonucleotides**		

Primer: F-18S: GCAAGACCGAAACTCAAAGG	Liu et al.^21^	N/A
Primer: R-18S: TGTTCATATGTCAAGGGCTGG	Liu et al.^21^	N/A
Primer: F-EF1a: AGCTTTACCTCCCAAGTCATC	Liu et al.^21^	N/A
Primer: R-EF1a: AGAACGCCTGTCAATCTTGG	Liu et al.^21^	N/A
Primer: 9087_RPS6A LP: AAAAGACATTG CAGTAAGGCG	This paper	N/A
Primer: 9088_RPS6A RP: AAATGGAAT CTGTACCTCGCC	This paper	N/A
Primer: 11264_NbU1small_F3: CCATCC TATTCCACCACCTCCT	This paper	N/A
Primer: 11265_NbU1small_R3: TTGAGA TTCCCTTCGTCAGTATTCG	This paper	N/A
Primer: 11266_NbU1small_F1: CCCTCC CGATGTCCCAGCCC	This paper	N/A
Primer: 11267_NbU1small_R1: TCCAG AACTTGCCCTTTGAGATTCCCTT	This paper	N/A

**Software and algorithms**		

*N. benthamiana* reference proteome (download from: https://nbenthamiana.jp/downloads)	Kurotani et al.^22^	Nbe_v1.1_pep.fa.gz
*N. benthamiana* genome annotation file (download from: https://nbenthamiana.jp/downloads)	Kurotani et al.^22^	Nbe_v1.1.2.sorted.fixed.gff3.gz
Perseus v.2.1.4.0 (download from: https://maxquant.net/perseus/)	Tyanova et al.^23^	N/A
R v.4.5.0 (download from https://www.r-project.org/)	R Core Team^24^	N/A
RStudio v.2025.05.0-496 (download from https://posit.co/download/rstudio-desktop/)	Posit Team^25^	N/A
Data repository (starprotocol_demo.zip; supplementary materials)	This paper	starprotocol_demo.zip

**Other**		

Aluminum foil	Any brand	N/A
Cell strainer, pore size: 100 μm, sterile	Sarstedt	Cat# 83.3945.100
Corning bottle-top vacuum filters (0.22 μm)	Corning	Cat# CLS430513
Filter pipette tips	Any brand	N/A
NanoDrop 2000 (or similar spectrophotometer)	Thermo Fisher Scientific	Cat# ND-2000
Pocket digital scale (to measure 0.1 g increases)	Any brand	N/A
Polypropylene Protein LoBind microcentrifuge 2 mL tubes	Eppendorf	Cat# 0030108.132
Refrigerated centrifuges for 50-, 15- and 2-mL tubes	Any brand	N/A
QuantStudio 3 real-time PCR system (or any qPCR system compatible with Sybr Green)	Thermo Fisher Scientific	Cat# A28567
Saran wrap	VWR	Cat# 129-3850
Stainless steel beads, size 4 mm	Sigma-Aldrich	Cat# BMSIPD96004M
Syringe (20 mL)	BD	Cat# 302830
Single-use syringe needle (G22)	BD	Cat# 305156
Thermal cycler	Any brand	N/A
Thermomixer or heating block with shaking	Any brand	N/A
UVP crosslinker CL-3000 (or similar)	Analytik Jena	Cat# 849-95-0615-02
Vortex	Any brand	N/A
Water bath	Any brand	N/A

## Materials and equipment

### Preparation of buffer stocks

Ensure all buffers are prepared in advance, unless otherwise indicated. Suggested preparation volumes are provided in parentheses.

**CRITICAL:** Ensure that all the reagents employed are of high purity and stored under adequate conditions (refer to vendor’s SDS for safety and handling information).

• Lysis buffer (base) (1 L)

**Table:** 

Reagent	Final concentration
MOPS	100 mM
CTAB	2%
EDTA	20 mM
NaCl	1.4 M

  ○ Dissolve all components (in solid form) in deionized water (ddH_2_O) in a beaker and adjust the pH to 7.0.
  ○ Filter using a 0.22 μm bottle-top vacuum filter into a clean, autoclaved bottle containing a magnetic stir bar.
    ***Optional:*** Divide the buffer into aliquots of 250 mL.
  ○ Add diethyl pyrocarbonate (DEPC) to a final concentration of 0.2% and mix overnight using a magnetic stirrer.
  ○ Remove the magnetic stir bar and autoclave the solution at 121°C for at least 17 min to inactivate DEPC.
    ***Note:*** DEPC is inactivated at high temperatures, becoming harmless after heat treatment.
  ○ Store lysis buffer (base) for up to 6 months at 20–25°C protected from light.
    **CRITICAL:** MOPS is sensitive to light. To prevent oxidation, protect the container with aluminum foil or use light-opaque glassware.
    **CRITICAL:** Do not use the solution if it appears yellowish or contains precipitates.

• DEPC-treated water (2 L). Fill four 500 mL bottles with ddH_2_O and add DEPC to a final concentration of 0.1%.
  ○ Mix thoroughly and incubate for at least 2 h at room temperature.
  ○ Sterilize by autoclaving.
  ○ DEPC-treated water can be stored indefinitely at 20–25°C.
• 1 M Tris pH 8.0 (500 mL). Dissolve Tris in DEPC-treated ddH_2_O to a final concentration of 1 M.
  ○ Adjust the pH to 8.0 using HCl.
  ○ Sterilize by autoclaving.
  ○ Tris can be stored indefinitely at 20–25°C.
• 0.5 M EDTA (250 mL). Dissolve EDTA in DEPC-treated ddH_2_O to a final concentration of 0.5 M.
  ○ Adjust the pH to 8.0 using NaOH pellets.
  ○ Sterilize by autoclaving.
  ○ EDTA can be stored indefinitely at 20–25°C.

***Note:*** EDTA is only soluble in water when the pH is adjusted to a basic range, typically pH 8.0.

• 5 M NaCl (250 mL). Dissolve NaCl to a final concentration of 5 M.
  ○ Treat with 0.2% DEPC.
  ○ Sterilize by autoclaving.
  ○ NaCl can be stored indefinitely at 20–25°C.
• 50× Complete protease inhibitor. Dissolve one tablet in 1 mL of 10 mM Tris (pH 8.0). This solution can be stored for up to 12 weeks at −20°C.
• TE-SDS 0.5% (100 mL). Prepare a solution containing 10 mM Tris (pH 8.0), 1 mM EDTA (pH 8.0) and 0.5% sodium dodecyl sulfate (SDS) from sterile stocks.
  ○ Treat with 0.2% DEPC and mix thoroughly.
  ○ Inactivate DEPC by incubating the solution overnight at 37°C.
  ○ This solution can be stored indefinitely at 20–25°C.

***Note:*** DEPC treatment is ineffective for concentrated Tris solutions and is only effective at low concentrations.

• 3 M sodium acetate (pH 5.2) (100 mL). Dissolve sodium acetate in ddH_2_O to a final concentration of 3 M and adjust the pH to 5.2 using glacial acetic acid.
  ○ Treat with 0.2% DEPC.
  ○ Sterilize by autoclaving.
  ○ This solution can be stored indefinitely at 20–25°C.
• 0.2 M Sodium citrate buffer (pH 6.0) (50 mL). Dissolve sodium citrate salt to 0.2 M in ddH_2_O and adjust the pH to 6.0 with HCl.
  ○ Treat with 0.2% DEPC.
  ○ Sterilize by autoclaving.

**CRITICAL:** Store protected from light at 4°C.

• 70% and 80% ethanol. Dissolve molecular biology-grade ethanol in sterile DEPC-treated water to the desired concentration.
  ○ Solution can be stored for up to one month in an airtight container at 20–25°C.
• 2× RNA fragmentation buffer (50 mL). Prepare a solution containing 20 mM Tris (pH 8.0) and 50 Mm MgCl_2_ from sterile stocks.
  ○ Treat with 0.2% DEPC and sterilize by autoclaving.
  ○ This solution can be stored for up to one month at 4°C.
• 100 mM PMSF. Dissolve PMSF in isopropanol to a final concentration of 100 mM. Prepare aliquots of 0.5 mL and store at −20°C for up to six months.
• 50 mM TEAB. Prepare the required amount immediately before use from a 1 M stock in UltraPure water.

**Table:** 2× Proteinase K reaction buffer (50 mL)

Reagent	Final concentration
Tris (pH 8.0)	50 mM
EDTA (pH 8.0)	40 mM
SDS	4%
NaCl	250 mM

Store for up to one month at 20–25°C protected from light.

  ○ Mix all the components from sterile stocks.
  ○ Treat with 0.2% DEPC and mix well.
  ○ Inactivate DEPC by incubating the solution overnight at 37°C.

### Installation of software and dependencies

• Download and install Perseus (v.2.1.3.0) or the latest version.
  ○ Place the extracted folder on your desktop.
  ○ Open the Perseus folder and create a shortcut to the “StartPerseus” executable for quick access and move it to the desktop.
    ***Note:*** Installation on the desktop allows editing Perseus files without administrator privileges.
  ○ Open Perseus and install any required dependencies, following on-screen instructions.
    ***Note:*** This step is only required the first time the program is run.

• Download and install R (v.4.5.0) or the latest version.

***Optional:*** Download and install RStudio (v.2025.05.0-496) or the latest version.

## Step-by-step method details

### Tissue harvesting and *in vivo* UV crosslinking

**Timing: 3 h**

In this step, the plant tissue is harvested and RNA-protein complexes are crosslinked *in vivo* using UV light (254 nm wavelength).

1. Prepare a tray with ice (sized to fit inside the UV crosslinker) and set the crosslinker’s energy setting to 375 mJ/cm^2^.

**CRITICAL:** The amount of UV light depends on the UV-crosslinker model, the type of plastic wrap and the tissue. It must be experimentally determined using a dose-response curve. Troubleshooting 1.

**Figure 2:**
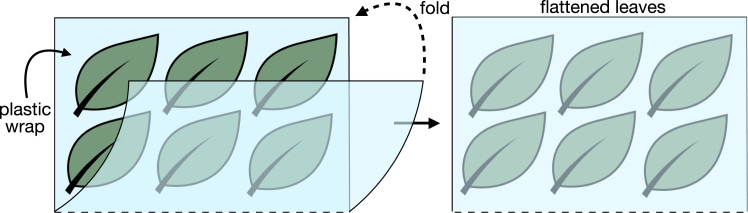
Diagram illustrating the arrangement of leaves for UV crosslinking Leaves are placed flat on plastic Saran wrap with the adaxial surface facing upward. The wrap is then folded to cover the leaves, ensuring they remain flattened and allowing alternating UV irradiation on both sides.
4. Crosslink the tissue.
  a. Place the envelope on the ice tray with the adaxial surface facing up.
  b. Insert it into the UV-crosslinker and begin irradiation.
  c. Allow the leaves to recover for 30–60 s, then flip the envelope and irradiate the abaxial surface.
  d. After recovery, irradiate the adaxial surface once more to enhance crosslinking.

***Note:*** Up to three leaf envelopes can be processed simultaneously by alternating irradiations. The recovery time for one envelope is the irradiation time of the next.

2. Harvest the two uppermost leaves from enough plants to obtain 5 g of tissue (typically, 20 leaves are sufficient).
3. Prepare the leaves for crosslinking.
  a. Cut a piece of Saran wrap of approximately 50 cm in length.
  b. Arrange the leaves with the adaxial surface facing up on the wrap.
  c. Fold the wrap to cover and flatten the leaves, forming an envelope (Figure 2).

**CRITICAL:** Ensure that nothing obstructs the UV sensor inside the crosslinker to avoid inconsistencies in UV exposition.

5. Unwrap the envelope and rewrap the leaves with aluminum foil.
  a. Label with sample name and date.
  b. Snap-freeze the wrapped leaves in liquid nitrogen.
  c. Repeat for all the samples.
6. Grind the frozen tissue to a fine powder and transfer to a sterile 50 mL tube.

**Pause point:** Samples can be stored for at least 3 months at −80°C until further processing.

### Tissue lysis and phase separation

**Timing: 4 h**

In this section, plant tissue is lysed and phenol-chloroform phase separation is performed to isolate the RNA-protein crosslinked complexes from the interphase.

***Note:*** All the steps must be conducted in a fume hood to avoid exposure to hazardous solvents.

**CRITICAL:** From this point onwards, all plasticware must be polypropylene to ensure compatibility with phenol and chloroform.

7. Preheat the water bath to 65°C and chill centrifuges for 50 mL, 15 mL and 2 mL tubes to 4°C.
8. Prepare the required volume of lysis buffer (approximately 8 mL per sample).
  a. To the Lysis buffer base, add the following to reach final concentrations: 3% PVPP, 1% 2-mercaptoethanol and 1× Complete protease inhibitor cocktail.
  b. Incubate the buffer in a water bath at 65°C for at least 10 min.
  c. Just before use, add PMSF to a final concentration of 1 mM.

***Note:*** This buffer must be freshly prepared. PVPP is insoluble and will make the solution milky. Mix the buffer thoroughly before use, as PVPP precipitates over time.

**CRITICAL:** PMSF is unstable in aqueous solution and should be added immediately before lysis.

9. Weigh 5 g of tissue powder into a cold 50 mL tube using a digital pocket scale. Re-freeze in nitrogen when needed.
  a. Allow the tissue to equilibrate at room temperature for 5 min inside the fume hood.

**CRITICAL:** Keep tissue frozen during handling.

**CRITICAL:** This protocol is optimized for 4–5 g or tissue. Using different amounts may require optimization.

10. Add 7.5 mL of pre-warmed lysis buffer and shake vigorously to fully resuspended the tissue.

**CRITICAL:** Manual shaking is more effective than using a vortex, as the solution becomes dense and viscous. Adding 4–5 steel beads (⌀4 mm) can enhance tissue homogenizing.

***Note:*** A skilled user can process up to eight samples in parallel.

11. Incubate the tubes in a 65°C water bath for 30 min and shake manually every 5 min.

**CRITICAL:** Ensure that tubes remain submerged during incubation to guarantee the temperature.

12. After lysis, cool the tubes to room temperature for 5 min.
13. Centrifuge at 4,000 × *g* for 30 min at 4°C to clear the lysate.

**CRITICAL:** If incomplete lysis is observed, adjust the volume of lysis buffer employed in step 11. Troubleshooting 2.

14. Filter the supernatant through a cell strainer (100 μm nylon mesh) into a new 50 mL tube.
15. Collect lysate to prepare input samples (non-crosslinked only).
  a. Transfer 500 μL of lysate from non-crosslinked samples into a 2 mL microcentrifuge tube.
  b. Add 1.5 mL of methanol to precipitate proteins and store at −20°C until use.
16. Add 3 M sodium acetate pH 5.2 to all the lysates (crosslinked and non-crosslinked) to reach a final concentration of 100 mM.
17. Add one half volume of water-saturated phenol to all the samples and mix by vortex for 5 s.

**CRITICAL:** Use acidic phenol (pH ≤ 6.0) to protect RNA integrity.

18. Add one half volume of chloroform and vortex for 10 s. Incubate at room temperature for 5 min.

19. Centrifuge at 4,000 × *g* for 30 min at 4°C to separate phases.
  ***Optional:*** Collect aqueous phase to isolate RNA for quality check and for other downstream applications.
  a. Transfer 1 mL of the aqueous phase (all samples) into a new 2 mL tube for RNA isolation.
  b. Add 120 μL of 5 M NaCl and 880 μL of isopropanol. Mix thoroughly and store at −20°C. For long-term storage (up to 1 year), keep at −80°C.

20. Carefully remove and discard the remaining aqueous (top) and organic (bottom) phases using a syringe with a G22 needle. Avoid disturbing the interphase.

***Note:*** The interphase contains the crosslinked RNA-protein complexes.

21. Vortex the interphase until homogenized and transfer it into a new 15 mL centrifuge tube.
22. Centrifuge at 12,000 × *g* for 5 min at 4°C.
23. Remove all residual aqueous and organic phases.
24. Transfer the interphase to a new 2 mL tube and fill with Trizol LS to the 1.5 mL mark.
  a. Vortex thoroughly to resuspend.

**Pause point:** Samples can be stored at −20°C for up to 1 month before continuing. Use this pause point to synchronize extractions.

### Interphase washing and removal of chromatin traces

**Timing: 5 h**

In this section, interphases containing RNA-protein complexes are washed to remove contaminants, including non-RNA-associated proteins and chromatin.

**CRITICAL:** Use filter pipette tips from this point onwards.

25. Before beginning, pre-cool a centrifuge for 2 mL tubes to 4°C.

***Note:*** All centrifugation steps are performed at 4°C.

26. Thaw the interphases resuspended in Trizol LS (from the step 25) and vortex thoroughly.
27. Add 500 μL of chloroform and shake manually for 30 s.
  a. Incubate at room temperature for 5 min.

***Note:*** A skilled user can handle up to 12 samples at a time. Handling more will increase variation in incubation times.

28. Centrifuge 10 min at maximum speed (preferably >15,000 × *g*).
29. Carefully remove and discard the aqueous and organic phases using a syringe (G22 needle), leaving the interphase intact.

***Note:*** Work quickly to minimize RNA degradation.

30. Add 150 μL of 1 mM sodium citrate buffer pH 6.0 to the interphase and vortex thoroughly.
31. Add 750 μL of Trizol LS and 250 μL of chloroform.
  a. Shake manually for 30 s.
  b. Incubate for 3 min at room temperature.
32. Centrifuge for 3 min at maximum speed.
33. Repeat the wash steps (30 to 33) twice for a total of 3 extractions.

**CRITICAL:** If the interphase becomes diffuse during phase removal, vortex thoroughly and centrifuge again to recompact it. Troubleshooting 3.

***Note:*** Interphases from non-crosslinked samples may appear diffuse or minimal due to the absence of RNA-protein complexes.

34. After the third wash, discard both the aqueous and organic phases, leaving the interphase intact.
35. Add 300 μL of TE-SDS 0.5% buffer to the interphase and vortex thoroughly.
36. Fill the tubes with methanol and vortex again to disrupt the interphases and precipitate proteins together with associated RNA.
37. Incubate at −20°C overnight (or for at least 4 h).

**CRITICAL:** Do not store tubes at −80°C, as this may cause proteins to adhere to the tube walls, reducing recovery.

**Pause point:** Samples can be stored at −20°C for at least 1 week before proceeding.

38. Centrifuge 30 min at maximum speed at 4°C to pellet the RNA-associated proteins.
39. Carefully decant the supernatant and wash the pellet with 1 mL of 70% ethanol.
  a. Vortex and centrifuge for 5 min.
40. Dry the pellet.
  a. Decant the ethanol.
  b. Spin-down the tubes for 30 s and use a pipette to remove any remaining ethanol.
  c. Air-dry the pellet in a flow hood for exactly 10 min.

**CRITICAL:** Ethanol residues inhibit DNase I activity, while over-drying may make the pellet difficult to resuspend. Troubleshooting 4.

41. Add 305 μL of UltraPure water (see key resources table) and vortex gently until pellet releases. Incubate on ice for 15 min to allow hydration.
42. For each sample, prepare a DNase I mix by combining 10 μL of DNase I with 35 μL of 10× DNase buffer in a new tube. Keep on ice.

**CRITICAL:** DNase I is sensitive to mechanical stress. Avoid vigorous pipetting or resuspension using a vortex.

43. Resuspend the pellets by gently pipetting up and down with a 1 mL pipette tip, breaking large fragments into smaller pieces.

**CRITICAL:** Avoid extended pipetting to prevent heat buildup and mechanical RNA fragmentation.

44. Add 45 μL of DNase I mix to each tube and mix gently by flicking the tubes.
45. Incubate at 37°C for 1 h to degrade residual DNA.
46. Stop the reaction by adding 750 μL of Trizol LS and 250 μL of chloroform.
  a. Vortex thoroughly.
  b. Incubate at room temperature for 3 min.
47. Centrifuge at maximum speed for 3 min.

***Note:*** At this point, DNase I and degraded nucleic acids partition into the aqueous and organic phases, respectively, while intact RNA-protein complexes remain in the interphase.

48. Carefully remove the aqueous and organic phases, preserving the interphase.
49. Fill the tubes containing the interphase with methanol to precipitate the RNA-protein complexes and remove residual Trizol.
50. Store at −20°C overnight.

**Pause point:** Samples can be stored at −20°C for at least 1 week before continuing.

### Elution and purification of RNA-bound proteins

**Timing: 5 h**

In this section, RNA-bound proteins are released by RNA degradation and subsequently precipitated for downstream analysis.

51. Centrifuge the methanol-precipitated samples (from the previous step) at maximum speed for 30 min at 4°C to pellet the complexes.
  a. During centrifugation prepare the required amount of 2× RNA fragmentation buffer.
52. Carefully decant the supernatant.
53. Wash the pellets once with 70% ethanol.
  a. Remove residual ethanol.
  b. Allow the pellets to air-dry, as described in previous steps.
54. Resuspend the pellets in 300 μL of UltraPure water.
  a. Vortex gently to detach the pellets.
  b. Allow complete hydration by incubating at 4°C for 15–30 min and occasional swirling using a vortex.

***Note:*** During pellet hydration, preheat a heat block to 95°C.

55. Prepare the samples for RBP release by RNA degradation.
  a. Divide the resuspended interphases into two 150 μL aliquots: use one for the following steps and store the other at −20°C as a backup.
    **CRITICAL:** If RNA will be analyzed (e.g., RT-qPCR or RNA-seq), use the backup sample for RNA purification (steps 84a-i to 89).
  b. Add 150 μL of 2× RNA fragmentation buffer and mix thoroughly.

56. Incubate at 95°C for 7 min in a heat block to fragment the RNA chemically.
  a. Cool down the samples to room temperature for 2 min.
57. Add 10 μL of RNase A/T1 mix and vortex thoroughly.
  a. Incubate at 37°C for 3 h to complete RNA degradation.

**CRITICAL:** RNase A/T1 will remain in the sample and it is typically among the most abundant proteins detected by LC-MS/MS. Do not increase the enzyme concentration. Troubleshooting 5.

58. Stop the reaction by adding 300 μL of Trizol LS and 100 μL of chloroform.
  a. Vortex thoroughly.
  b. incubate for 3 min at room temperature.
59. Centrifuge at maximum speed for 5 min to separate phases.
60. Carefully tilt the tube and use a 200 μL pipette to extract 190 μL from the bottom of the organic phase. This contains the proteins released from RNA after digestion.

**CRITICAL:** Tilting the tube displaces the interphase, allowing more precise access to the organic phase. Avoid contaminating the sample with interphase, which may carry glycoproteins and other impurities.

**CRITICAL:** Transfer the 190 μL of organic phase containing the released RBPs into a polypropylene Protein LoBind 2 mL tube.

61. To precipitate the RBPs, add 1.8 mL methanol and incubate overnight at −20°C.

**Pause point:** Samples can be stored at −20°C for up to one week prior to further processing.

### Resuspension of (RNA-binding) proteins

**Timing: 1.5 h**

In this section, proteins from both input samples and interphases are cleaned, concentrated, and prepared for downstream analysis such as SDS-PAGE, western blot, or LC-MS/MS.

62. Centrifuge the protein input samples (from step 15) and the precipitated RBPs (from step 63) at maximum speed for 30 min at 4°C to pellet the proteins.
63. Wash the pellets once with 80% ethanol, followed by a second wash with 70% ethanol.
  a. Decant the ethanol and allow the pellets to air-dry as described earlier.

***Note:*** Residual Trizol in the samples may lead to overestimation of protein concentration in colorimetric assays such as BCA and Bradford.

64. Resuspend the input pellets in 800 μL of 50 mM TEAB (or buffer of choice) and the RBP pellets in 50 μL.
  a. Allow the pellets to hydrate at 42°C for 10 min. If possible, use a thermomixer or heat block with shaking at >1000 rpm to facilitate resuspension.

***Note:*** For LC-MS/MS analysis, we recommend resuspending the proteins in 50 mM triethylammonium bicarbonate (TEAB), as this is compatible with multiplex labelling MS techniques. However, confirm the required buffer composition with your proteomics service provider. The end state of samples here is compatible with most proteomics sample preparation approaches commonly used.

65. Prepare samples for LC MS/MS analysis following the instructions of your provider.

### Analysis of proteomics data

**Timing: 1.5 h**

In this section, the mass spectrometry data generated using the samples produced at the end of the previous section, will be analyzed using Perseus software^23^ to identify RBPs enriched in crosslinked fractions. After running the provided R script, the user will generate two volcano plots comparing the enriched proteins before and after filtering.

***Note:*** Mass spectra are first processed with MaxQuant^26^ (or a similar proteome database search engine) to perform peptide-spectrum matching and quantification. The *N. benthamiana* reference proteome^22^ (see key resources table) is used for protein identification. Confirm with your proteomics provider the type and format of output files you will receive. Perseus is highly compatible with the Maxquant search engine used here but may be less optimal when using other database search engines.

**CRITICAL:** For the demonstration script to run without modifications, retain the file names provided in the supplementary materials and in this section.

66. After peptide-spectrum matching, a tabular file containing protein groups will be generated.
67. Download the provided data package (Data S1 - starprotocol_demo.zip) from the key resources table and extract it in your desired working directory.
68. Load the *N. benthamiana* annotation file on Perseus.
  a. Navigate to: Desktop > Perseus > bin > conf > annotations.
  b. Copy the file annot_nbenth_MPP_v7.txt into this directory.

***Note:*** The annotation file is a tab-separated values file and can be explored using spreadsheet software (e.g., Microsoft Excel).

**CRITICAL:** Ensure that the protein identifiers in your annotation and sequence database are compatible. If using custom proteomes, annotate them using tools like eggNOG-mapper.^27^

69. Load the proteinGroups.txt file in Perseus:
  a. Go to: Matrix > Generic matrix upload.
  b. Select the columns containing the LFQ values from the list (18 columns labeled “LFQ intensity…”).
  c. Move them to the “Main” section pressing the “>” button.
  d. Press OK to generate the matrix for analysis.
70. Clean-up the matrix:
  a. Remove the following rows using: Filter rows > Filter rows based on categorical column.
    i. Potential contaminants.
    ii. Reverse hits.
    iii. Proteins identified only by site.

***Note:*** In the provided dataset, this will reduce the example data from 4309 to 4068 entries.

71. Group the biological replicates by sample type:
  a. Go to: Annot. rows > Categorical annotation rows.
  b. Select: “Read from file” and choose the provided file grouping_fraction.txt.

***Note:*** Alternatively, annotations can be done manually by selecting replicates and assigning a group name.

72. Filter the identified proteins for reproducibility:
  a. Apply: Filter rows > Filter rows based on valid values.
  b. Select the option “Percentage” and set it to 70.
  c. Select “In at least one group”, to keep only proteins present in ≥5/6 replicates, in at least one fraction.
73. Add annotations to the identified protein groups:
  a. Go to: Annot. columns > Add annotation.
  b. Select the annot_nbenth_MPP_v7.txt file and import desired metadata. For demo purposes, load all the listed annotations.
74. Log2 transform the LFQ data.
  a. Apply: Basic > Transform.
75. Impute missing values to allow statistical comparisons:
  a. Apply: Imputation > Replace missing values from normal distribution. Use default settings.

***Note:*** The matrix generated at this point can be analyzed using the software of preference (e.g., R).

76. Perform statistical testing to identify enriched proteins:
  a. Go to: Tests > Two-sample test.
  b. Assign “OOPS_XL” to the group 1 (top) and “OOPS_NOXL” to the group 2 (bottom).
  c. Select “Welch’s T-test”.

**CRITICAL:** A non-parametric test is employed due to missing values in non-crosslinked samples.

77. Export the results of the Welch’s *t* test.
  a. Go to: Export > Generic matrix export.
  b. Save the new matrix as starprotocol_rbpome.txt.

78. Filtering enriched RBPs.
  a. Open starprotocol_rbpome.txt in a spreadsheet program.
    **CRITICAL:** Delete the second row (Perseus metadata), which can interfere with downstream analysis.
  b. Filter the column “Welch’s T-test Significant OOPS_XL_OOPS_NOXL” to retain only “+” entries. This will reduce the dataset from 4068 to 431 protein groups.
  c. Apply a second filter on “Welch’s T-test Difference OOPS_XL_OOPS_NOXL” to retain proteins with a fold change ≥1. This yields 369 significantly enriched RBPs.
  d. Save the filtered list as starprotocol_enriched_RBPome.txt.

79. Visualization of enriched RBPs (volcano plot) using RStudio.
  a. Ensure that both the starting matrix (starprotocol_rbpome.txt) and the filtered matrix (starprotocol_enriched_RBPome.txt) are in the same folder.
  b. Run R on the terminal or open RStudio.
  c. Set the provided script volcano_rbpome.R.
    i. Update the section “## 0. Set working directory” with the location of your files.
    ii. Save changes.
  d. In RStudio, select all the lines and run the script to generate a volcano plot.
    i. A new figure will be automatically stored in your working folder (volcano_rbps_starprotocol.pdf) showing data before and after filtering.

***Note:*** If working with custom data, update the file names in section “## 2. File paths of the script” accordingly:

file_unf <- "starprotocol_rbpome.txt"

file_flt <- "starprotocol_enriched_RBPome.txt"

***Optional:*** If graphical user interface is preferred, the volcano plots can be generated in Perseus using the matrix created in step 75, selecting OOPS_XL as the first group and OOPS_NOXL as the second group.

80. Use the list of enriched RBPs (step 77) for downstream analyses, such as those performed in this publication, as well as for validations or hypothesis generation.

**CRITICAL:** Perform quality checks on the mass spectrometry data to ensure the enriched proteins are true RBPs (see expected outcomes section). Troubleshooting 6.

### Purification of high-quality RNA with DNase and proteinase K

**Timing: 6 h**

In this section, high-quality RNA is isolated for downstream applications such as electrophoresis, RT-qPCR or RNA-seq.

81. Centrifuge the RNA-containing samples collected in step 19 for 15 min at max speed at 4°C to pellet the RNA.
82. Wash the resulting RNA pellet with 1 mL of 70% ethanol and vortex thoroughly.
83. Centrifuge for 5 min at maximum speed.
  a. Remove any residual ethanol and air-dry the pellet as previously described.
84. Resuspend the pellet in 500 μL of 1 mM sodium citrate (pH 6.0).
85. Quantify the RNA using a NanoDrop or equivalent spectrophotometer.

***Note:*** A260/A280 ratios are expected to be <2.0 due to proteins covalently bound to the RNA in crosslinked fractions. A260/A230 should be ∽2.2, indicating minimal salt and solvent contamination.

86. Purification of RNA with DNase and proteinase K.
  a. Transfer 30–50 μg of RNA to a new 2 mL tube. Store the remaining sample at −80°C.
    ***Optional:*** If using the interphase containing RNA-protein adducts (backups from step 56a), quantify the RNA using a NanoDrop and transfer 30–50 μg to a new 2 mL tube.
    ***Optional:*** For non-crosslinked interphase controls, use the same volume as the corresponding crosslinked fraction (these often yield minimal RNA).
  b. Adjust all sample volumes to 85 μL with UltraPure water.
    ***Note:*** Keep sample volumes minimal to maintain concentration.
  c. Prepare DNase I master mix by adding 5 μL of DNase I and 10 μL of 10× DNase buffer per sample. Mix gently.
  d. Add 15 μL of DNase I master mix to each RNA sample and mix gently by pipetting.
  e. Incubate at 37°C for 45 min.
    i. During DNase I treatment, prepare the required amount of 2× proteinase K reaction buffer.
  f. To stop the reaction, add 110 μL of 2× proteinase K reaction buffer.
  g. Add 10 μL of proteinase K to each sample and mix thoroughly.
  h. Incubate at 55°C for 1.5 h (use shaking at 550 rpm if available) to degrade the DNase and crosslinked proteins on the RNA.

87. After digestion, add: 660 μL of TE-SDS 0.5%, 120 μl of 5 M NaCl and 2 μl of glycogen.
  a. Vortex thoroughly.
88. Add 1 mL of isopropanol and mix thoroughly.
  a. Incubate at least two hours or overnight at −20°C to precipitate the purified RNA.
89. Centrifuge at maximum speed for 30 min at 4°C.
  a. Remove the supernatant.
90. Wash the pellet twice with 70% ethanol.
  a. Dry the pellet as described before.
91. Resuspend the RNA in 50 μl of 1 mM sodium citrate (pH 6.0).
92. Quantify by the preferred method.

### RNA analysis by RT-qPCR

**Timing: 5 h**

In this section, purified total RNA and deproteinated RNA are reverse transcribed into cDNA and analyzed via quantitative PCR to detect transcript abundance.

93. Reverse transcription (cDNA synthesis).
  a. Prepare the following mix to anneal primers for reverse transcription.
    **Table:** Mix for RNA priming

Reagent	Amount
RNA	0.5–2.5 μg∗
Random hexamers (50 μM)	1.0 μL
Oligo dT (50 μM)	0.5 μL
Water	To make 10 μL

    ∗**CRITICAL:** For non-crosslinked interphase controls, use the same volume as the corresponding crosslinked fraction (the RNA yield in these samples is minimal).
    **CRITICAL:** Random hexamers are required because this protocol isolates both polyadenylated and non-polyadenylated RNAs.
  b. Denature RNA-primer mix at 70°C for 5 min in a thermal cycler.
  c. Immediately transfer the tubes to ice to stabilize primer annealing.

94. Prepare a reverse transcription (RT) master mix:
  **Table:** Reaction mix for RT

Reagent	Amount (per sample)
Water	1.5 μL
5× RT buffer	4.0 μL
dNTPs (0.2 mM each)	2.0 μL
Ribolock RNase inhibitor	0.5 μL
Reverse transcriptase	1.0 μL

  a. Add 9 μl of RT master mix to each RNA-primer mix.
  b. Mix thoroughly and briefly spin down.
  c. Run the RT reaction in a thermal cycler using the following program:
    **Table:** RT program

Step	Temperature	Time
Primer annealing	25°C	10 min
Reverse transcription	42°C	60 min
Enzyme inactivation	85°C	5 min
  d. After the RT reaction, adjust the cDNA concentration to 25 ng/μl (of input RNA) using 10 mM Tris pH (8.0).
    **Pause point:** Store the cDNA at −20°C or −80°C for long-term until use.

95. Perform quantitative PCR using cDNA.
  a. Prepare a PCR master mix as follows (in this order):
    **Table:** qPCR master mix

Reagent	Amount (per sample)
Water	9.0 μL
HOT FIREPol EvaGreen qPCR Supermix (5×)	3.0 μL
Primer 1 (3 μM)	1.0 μL
Primer 2 (3 μM)	1.0 μL
  b. Add 14 μl of qPCR master mix to the corresponding tubes or plates.
  c. Add 1.0 μl of cDNA sample to each tube/well.
  d. Spin down for 1 min at 1000 × *g* at room temperature.
  e. Run real time PCR using the following cycling conditions:

**Table:** qPCR cycling protocol

Step	Temperature	Time	Cycles
Initial activation	95°C	12 min	1
Denaturation	95°C	15 sec	40 cycles
Annealing/extension	60°C (detection in Sybr Green channel)	60 sec	
Melt curve	N/A	N/A	N/A

**CRITICAL:** Perform a standard curve to determine primer efficiency, optimal cDNA concentration, and presence of PCR inhibitors (e.g., residual phenol or alcohol).

**Figure 3:**
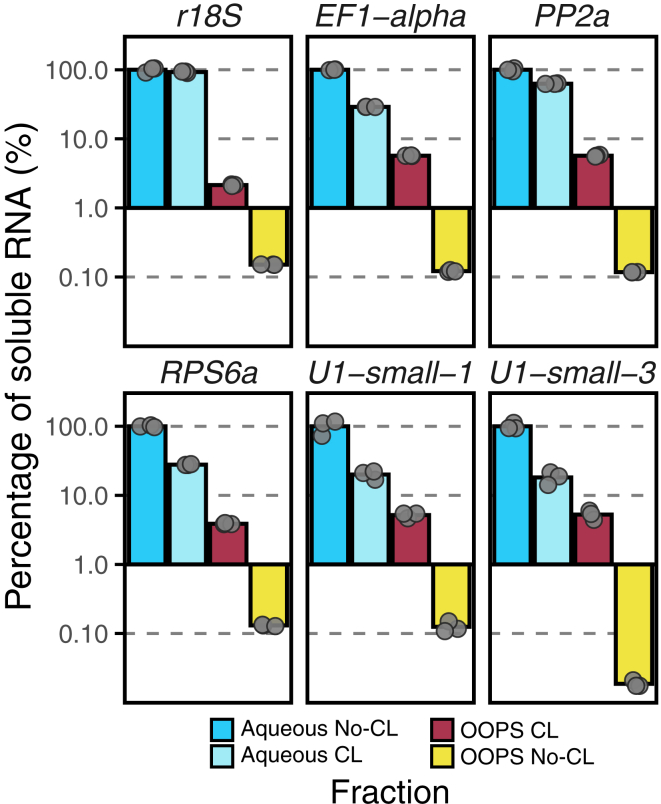
RT-qPCR analysis of RNA species across different fractions R18S (large ribosomal subunit); EF1-alpha, PP2a, and RPS6a (mRNAs); and U1-small-1 and U1-small-U3 (snoRNAs) were analyzed. The highest RNA levels are detected in the aqueous fraction of non-crosslinked (No-CL) samples, which reflects the total transcriptome. Following crosslinking, the RNA bound to proteins is redistributed to the interphase, resulting in a moderate decrease in RNA abundance. In crosslinked (CL) samples, the RNA-protein complexes accumulate in the interphase. In the absence of UV exposure, RNA levels in the interphase remain negligible.

96. Calculate relative transcript abundance in the different fractions using the ΔCt method.
  a. Normalize Ct values to the non-crosslinked aqueous phase, which serves as the reference (=1) (Figure 3). Use the following formula:

$$\%RNA=100\cdot 2^{-\left({Ct}_{fraction}-{Ct}_{Non-CLaq}\right)}$$

Variables.

*Ct*_*fraction*_ = averaged Ct values obtained using RNA from a given fraction.

*Ct*_*Non–CLaq*_ = averaged Ct values obtained using RNA from the aqueous phase of non-crosslinked samples.

***Note:*** If different amounts of input tissue or RNA for cDNA synthesis are used, values must be corrected accordingly.

**CRITICAL:** Internal normalization genes (e.g., housekeeping genes) cannot be used in this protocol due to the nature of *in vivo* UV crosslinking and fractionation-specific RNA enrichments. Expression comparisons are made only between fractions, not across genes or treatments.

## Expected outcomes

**Figure 4:**
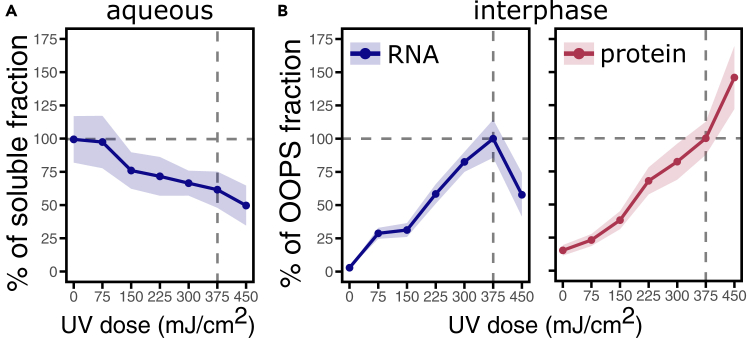
UV dose-response curve to determine optimal crosslinking conditions Yields of RNA and protein were measured after irradiation with increasing UV doses. (A) In the aqueous phase, RNA levels progressively decreased with increasing UV doses, likely due to crosslinking-induced relocation to the interphase. (B) In the interphase, RNA and protein yields increased proportionally with UV irradiation. RNA levels in the aqueous phase of No-CL samples were considered 100%, while OOPS fractions were normalized to a dose of 375 mJ/cm^2^.

To determine the optimal UV crosslinking conditions for *N. benthamiana* leaves, we performed a dose-response analysis. As shown in Figure 4, we applied incremental doses of UV irradiation (75 mJ/cm^2^ per step) to the leaves, followed by sample fractionation using the OOPS protocol. RNA and proteins were then isolated from the aqueous and interphase (OOPS) fractions, respectively, and quantified. We observed that the RNA yield in the aqueous phase progressively decreased with increasing UV doses. Conversely, both RNA and protein levels in the interphase increased, suggesting reduced RNA solubility, likely due to the stabilization of RNA-protein complexes upon UV light exposure, which resulted in their partitioning into the interphase. At the highest UV dose tested, we observed a decrease in RNA yield in the OOPS fraction, indicating possible degradation or over-crosslinking. Therefore, 375 mJ/cm^2^ was determined to be the optimal condition for efficient *in vivo* crosslinking of both stable and transient RNA-protein complexes in *N. benthamiana* leaves. We strongly recommend optimizing UV-crosslinking conditions according to tissue type, plastic film, and UV-crosslinker apparatus combination.

We provided the users with a proteomics dataset generated using this protocol, comprising six biological replicates. Two fractions were analyzed: the input and the OOPS interphase. Within the OOPS fraction, both crosslinked (CL) and non-crosslinked (No-CL) samples were processed, resulting in 18 samples analyzed. This experimental setup is essential for assessing RNA-binding protein enrichment.

**Figure 5:**
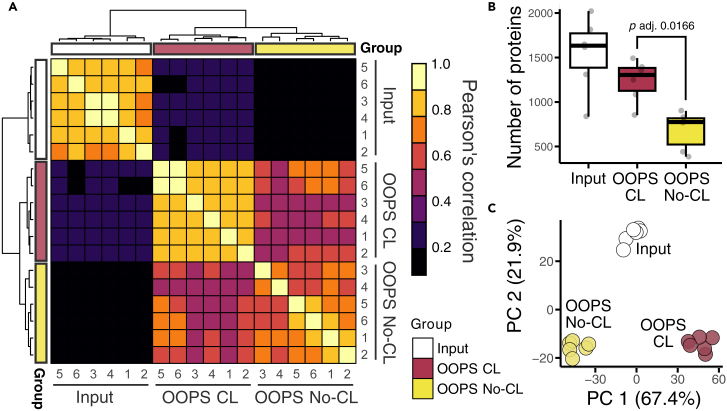
Quality control of the proteomics data from OOPS fractions of *N. benthamiana* leaves (A) Pearson correlation analysis of label-free quantification (LFQ) values across six replicates of input, OOPS CL, and OOPS No-CL samples. Clustering of similar sample types is displayed along the axes. (B) Protein group counts per fraction in the different samples. Significance was determined using Student’s *t* test. (C) Principal component analysis (PCA) reveals clustering of samples by fraction and crosslinking condition.

To ensure reproducibility, quality control analyses were conducted using Pearson correlation. As shown in Figure 5, the three sample groups clustered together, indicating high inter-replicate correlation. Additionally, we quantified the number of protein groups identified by LC-MS/MS. Consistent with previous results from our research group, the input fraction contained the highest number of protein groups, followed by the OOPS CL and OOPS No-CL fractions.

To further explore the dataset, we performed principal component analysis (PCA). Here, the replicates of each sample clustered together, in agreement with the correlation analysis. Additionally, PCA revealed that the primary source of variance (PC1 > 67%) was attributable to UV-crosslinking, followed by sample fractionation (PC2 > 21%). Collectively, these statistical analyses confirm that the protocol yields high-quality proteomics data.

**Figure 6:**
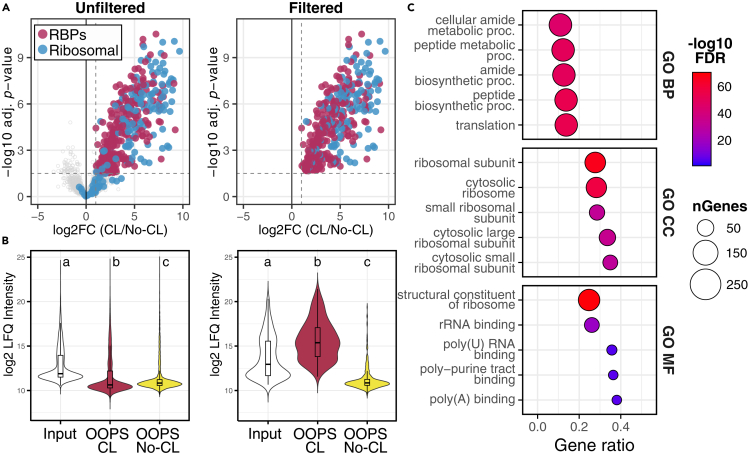
Enrichment analysis of RNA-binding proteins (RBPs) from *N. benthamiana* leaves (A) Volcano plots showing RBP enrichment in OOPS CL versus OOPS No-CL samples (log2 fold change ≥ 1), before (left) and after (right) data filtering. Ribosomal proteins (blue) and other RBPs (maroon) are highlighted. Dashed lines indicate thresholds. (B) Log2 LFQ values of proteins displayed in (A). Within each panel, different letters indicate statistical differences (*p*<0.001, one-way ANOVA with Tukey’s *post hoc* test). (C) Gene Ontology (GO) analysis of the filtered dataset showing the top five enriched terms per category: BP (biological process), CC (cellular component), and MF (molecular function). Color scale indicates -log10 FDR; bubble size, protein count; gene ratio, category representation.

To identify the RNA-binding proteome (RBPome) of *N. benthamiana*, we performed enrichment analysis by comparing protein abundances between the OOPS CL and OOPS No-CL fractions. Proteins with a ≥ 2-fold increase (log2 FC > 1) and adj. *p* > 1.5 (-log10) were considered enriched. In Figure 6, a visual comparison of the datasets before and after filtering using these criteria is provided. After removing non-enriched proteins, a final set of 369 protein groups was retained. To benchmark the results, we examined the enrichment of bona fide RBPs, such as ribosomal proteins. A significant enrichment of ribosomal proteins in the OOPS CL fraction compared to the input (*p* = 2.2e-16, Fisher’s exact test) validated the ability of our protocol for isolating RBPs.

We hypothesized that if a given RBP is crosslinked to RNA and subsequently captured in the interphase, its abundance would be higher in this fraction compared to total lysates. To test this, we compared LFQ values across fractions, before and after filtering. As expected, unfiltered datasets showed the highest abundance in the input fraction. However, in the filtered dataset of enriched RBPs, we observed significantly higher abundance in the OOPS CL fraction compared to both the input and OOPS No-CL fractions (mean differences = +1.94 and +4.43 log2 LFQ, respectively; adj. *p* < 0.001, one-way ANOVA with Tukey HSD *post hoc* test), supporting the successful enrichment of RNA-associated proteins.

Following dataset validation, we assessed whether RNA-related functional categories were enriched in the RBPome. Gene ontology analysis was conducted using ShinyGO (v.0.82),^28^ revealing that the top five enriched terms in the categories of biological process, cellular component, and molecular function were associated with translation, ribosomal structures, and RNA binding, respectively. These results further confirmed the selective enrichment of RNA-binding proteins by our protocol.

**Figure 7:**
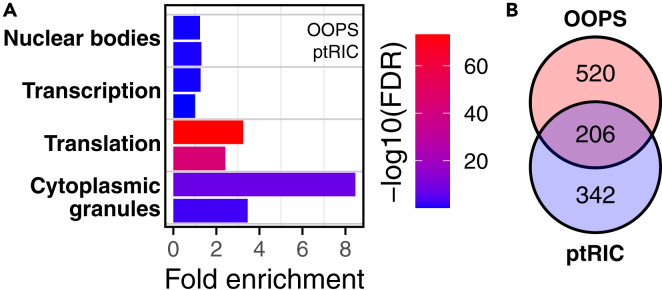
Comparison of RNA-binding proteomes (RBPomes) obtained by OOPS or by plant RNA-interactome capture (ptRIC) (A) Significantly enriched proteins present in both RBPomes were annotated and classified into relevant functional categories. For each category, fold enrichment is shown for OOPS (upper bar) and ptRIC (lower bar). Bar colors represent FDR values (-log10). (B) Number of proteins captured by each method. Unique and shared proteins are shown in the Venn diagram. For comparison, protein groups were expanded and individual protein IDs were counted.

To our knowledge, this is the first RBPome *of N. benthamiana* captured using phase separation. For benchmarking, results obtained with this protocol were compared with those generated using plant interactome capture (ptRIC) by Bach-Pages et al., 2020. Enrichment analysis for relevant functions and the number of identified proteins indicated that OOPS increased both enrichment and the number of detections (Figure 7).

**Figure 8:**
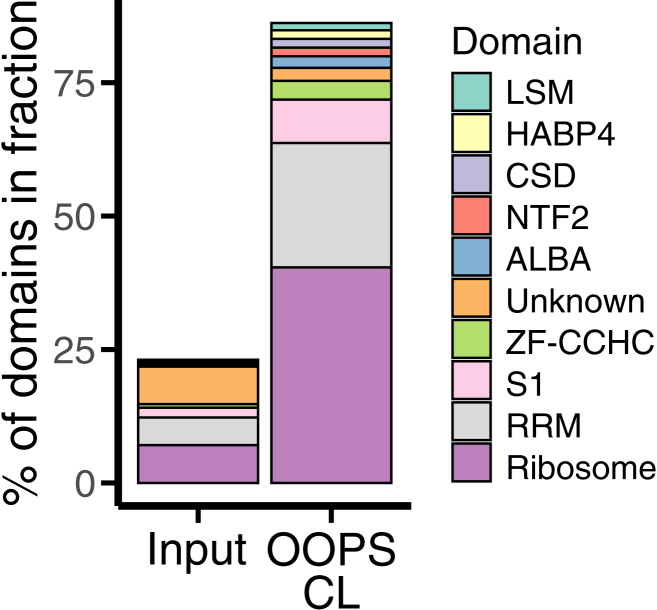
Protein domain analysis Proportions of the top nine protein domains identified in the OOPS UV crosslinked (CL) RBPome fraction compared to their representation in the total leaf proteome. Unknown, indicates proteins lacking annotated domains in the Pfam database.^29.^

Given that ribosomal proteins are highly expressed, we sought to rule out the possibility that their enrichment occurred by chance. We analyzed the top 10 enriched protein domains in the OOPS CL fraction and compared their distributions with the input fraction. In the OOPS CL fraction, domains related to ribosomal proteins, RNA recognition motifs (RRM), and S1 domains accounted for 40.4%, 23.3%, and 8.1% of proteins, respectively, compared to only 7.1%, 5.2%, and 1.8% in the input fraction (Figure 8). Overall, nine RNA-binding domains (RBD) constituted over 80% of all motifs in the OOPS CL fraction but less than 25% in the input, indicating the protocol enriched for a diverse set of RNA-associated domains and not only ribosome-related hits. In addition to RBDs, the “Unknown” category, comprising proteins without canonical RBDs,^11^ was also among the top enriched catagories (2.4%). Recent findings suggest that transient RNA associations may function as a regulatory mechanism for protein activity (riboregulation).^30^^,^^31^ For example, the activity of Enolase 1, a glycolytic enzyme, is reduced upon RNA binding,^32^ whereas enzymes such as GAPDH interact with RNA targets to regulate stability and translation (moonlighting activity).^33^ Although this is an emerging field, our protocol effectively isolates the leaf (stable and transient) RNA-binding proteome of *N. benthamiana*, generating high-quality datasets that can substantially contribute to advancing RNA-protein interaction studies in plants.

## Limitations

This protocol was established using young *N. benthamiana* leaves, a relatively simple organ that lacks specialized modifications such as lignification and is characterized by its thin, flat tissue structure. We acknowledge that adjustments may be required to optimize UV-crosslinking conditions for other tissues or species. One potential adaptation, as reported by others, is to perform UV irradiation on frozen, ground tissue.^34^

In addition, there are multiple experimental strategies available to capture RNA-binding proteomes. As shown in Figure 7, different methodologies enrich for specific proteins (and potentially distinct functions), as suggested by the non-overlapping proteins between OOPS and ptRIC.^11^ We therefore consider these approaches complementary, and the choice of method ultimately depends on the specific objectives and requirements of the user.

## Troubleshooting

### Problem 1

Low yield of RNA or RNA-binding proteins in the interphase.

### Potential solution

• Ensure that tissue crosslinking conditions (step 1) are optimized by performing a dose-response experiment, as exemplified in Figure 4. Quantify RNA and protein yields in the aqueous phase and in the interphase to assess optimal crosslinking efficiency.
• Refer also to Troubleshooting 2, as incomplete lysis can compromise RNA/protein recovery.

### Problem 2

Incomplete tissue lysis.

### Potential solution

• If incomplete lysis is suspected, increase the tissue-to-lysis buffer ratio in step 11. The protocol uses a 1:1.5 (weight/volume) ratio. We have tested 1:2 and 1:2.5 ratios without compromising yield or lysis efficiency. However, increasing the buffer requires proportional adjustments of sodium acetate, phenol and chloroform volumes during phase separation in steps 16–17 (see main protocol).
• Alternatively, increase the shaking frequency and/or intensity during lysis incubation (step 12) to enhance mechanical disruption of the tissue.

### Problem 3

Poor compacting of the interphase during washing steps.

### Potential solution

• Increasing centrifugal force significantly improves the interphase integrity during re-extractions (steps 26–32). We routinely use 20,000 × *g*; however, the protocol showed to be effective when 15,000 × *g* was used.
• Extended incubations before phase removal can result in diffuse interphases. If this occurs, vortex the samples thoroughly and centrifuge again. To minimize this issue, reduce the number of samples processed in parallel to shorten handling times.
• If the issue persists despite repeat centrifugation, inefficient crosslinking may be the underlying cause (see troubleshooting 1).

### Problem 4

Pellets containing RNA, proteins or RNA-protein complexes are difficult to resuspend (steps 39, 51, 63, 83 and 89).

### Potential solution

• Methanol and isopropanol precipitation lead to sample dehydration, making pellets difficult to dissolve. Complete removal of alcohol is essential for effective resuspension and downstream enzymatic treatments. After centrifugation:
  ○ Decant the supernatant and centrifuge briefly to collet residual droplets.
  ○ Use a pipette to remove any remaining alcohol.
  ○ Air-dry the pellet.

**CRITICAL:** Avoid over-drying the pellet, as this may be not reversible. Based on experimental optimization, a drying period of 10 min in a flow cabinet is sufficient to eliminate ethanol without over-drying.

### Problem 5

Low number of proteins enriched in the OOPS CL fraction.

### Potential solution

**Figure 9:**
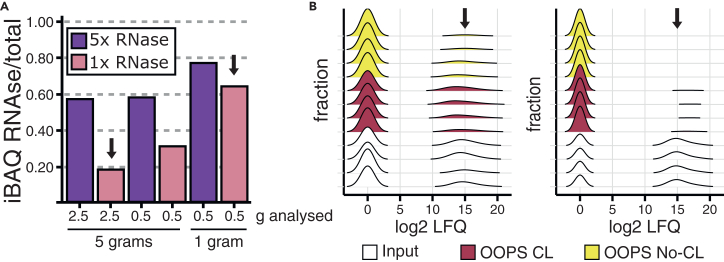
Optimization of tissue input and RNase ratio for proteomic analysis (A) LC-MS/MS analysis of OOPS CL samples from 5 g or 1 g of input tissue. Interphase fractions (equivalent to 2.5 g or 0.5 g) were treated with 1× or 5× RNase. Under optimal conditions (5 g, 1× RNase), RNase iBAQ signal was <30% while in 1 g samples, it exceeded 60%, compromising RBP detection. (B) LFQ intensity distribution across fractions from four CL and four No-CL samples, prepared using 5 g (2.5 g interphase, left) or 1 g (0.5 g interphase, right). Input samples showed similar intensities for both experimental designs. However, LFQ values from the OOPS fractions using 5 g were higher (indicated with arrows), consistent with the observations in panel (A). Peaks at zero correspond to imputed values.
  ○ LFQ intensity and total protein identifications decreased significantly (Figure 9). We hypothesize this is due to a molecular crowding effect mediated by RNA,^35^ which may enhance RNA-protein adducts partition and accumulation at the interphase. Reduced RNA concentrations in low-input samples may therefore impair RBP enrichment and detection.
• If low tissue input is required, we recommend scaling down RNase treatment proportionally.

• The protocol was optimized using 5 g of tissue input, with mass spectrometry performed using 150 μl of the resuspended interphase (equivalent to 2.5 g input; step 52a). The ratio between starting material and number of interphase re-extractions must be balanced to ensure sufficient RBP enrichment (see also troubleshooting 6).
• To evaluate the protocol at lower input amounts, we tested 1 g of tissue and analyzed either i) half of the corresponding interphase (0.5 g equivalent), or ii) 0.5 g-equivalent interphase obtained from a 5 g input. We identified two key limitations in low-input conditions:
  ○ The RNase A/T1 concentration to release RBPs must be adjusted relative to the interphase amount. At standard conditions (2.5 g input and 1× RNase mix), RNase peptides comprised ∼20% of total iBAQ intensity (Figure 9). In contrast, for 1 g input with the equivalent RNase concentration, RNase accounted for up to 70%, limiting detection of enriched RBPs to only few hits.

### Problem 6

High proportion of non-RNA-associated proteins are identified in the crosslinked (CL) OOPS fraction.

### Potential solution

**Figure 10:**
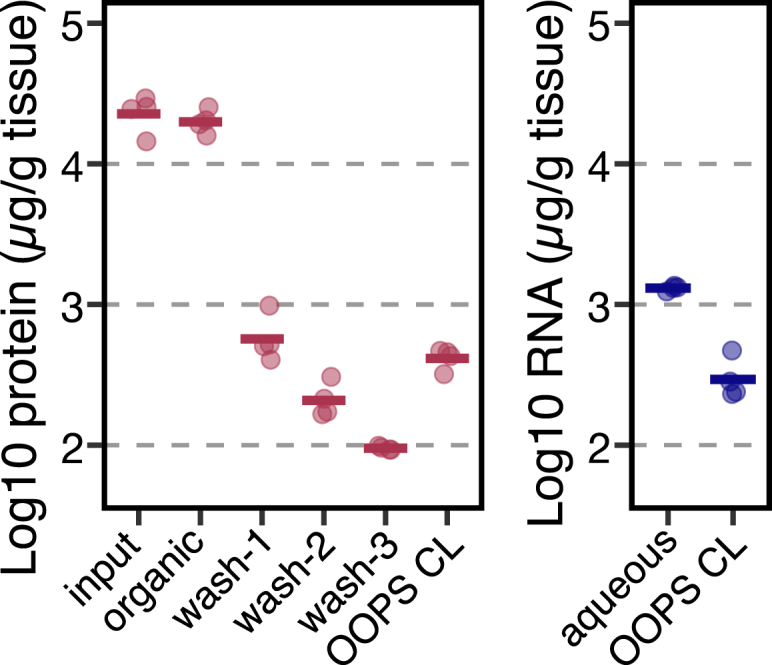
RNA and protein yield across the OOPS protocol Protein and RNA were quantified throughout the protocol, from the initial lysate, organic phases during washes and the final OOPS CL fraction. Protein release from the interphase to the organic phase decreased progressively through washing steps, while the yields remained relatively high after three washes in the interphase (left panel). RNA yields in the final OOPS CL fraction were comparable to protein (right panel).
• As an alternative, perform LC-MS/MS analysis of OOPS CL fractions after 2 to 6 re-extractions to determine the optimal number of washes.

• This issue may arise from residual contamination due to incomplete removal of non-crosslinked proteins. We recommend increasing the number of re-extractions to improve interphase purity. Assess wash efficiency as follows.
  ○ Precipitate 300 μl of the organic fraction with methanol (steps 59 to 63) after each re-extraction and measure the protein concentration.
  ○ Normalize protein yield to the total volume of the organic fraction.
  ○ A progressive decline in protein content across washes is expected. Re-extraction is considered sufficient when protein yield is higher in the OOPS CL fraction than in the organic phase (see Figure 10).

## Resource availability

### Lead contact

Further information and requests for resources and reagents should be directed to and will be fulfilled by the lead contact, Harrold van den Burg (h.a.vandenburg@uva.nl).

### Technical contact

Technical questions on executing this protocol should be directed to and will be answered by the technical contact, Victor A. Sánchez-Camargo (v.a.sanchezcamargo@uva.nl).

### Materials availability

This study did not generate new unique reagents.

### Data and code availability

The datasets generated during this study are available at ProteomeXchange: PXD068386.

## Acknowledgments

This research was supported by a Topsector T&U project (LWV20.105). We acknowledge Petra Houterman and Bas Beerens for technical assistance and Harold Lemereis and Ludek Tikovsky for growing and caring the plants used in this research.

## Author contributions

V.A.S.-C. designed and performed the experiments, analyzed and interpreted the data, and wrote the manuscript. G.K. contributed to the design of the proteomics experiments, performed LC-MS/MS sample analysis, participated in data interpretation, and reviewed the manuscript. H.A.v.d.B. conceived the research line, secured funding, contributed to experimental design and data interpretation, and critically reviewed the manuscript.

## Declaration of interests

H.A.v.d.B received funding from the industry for this study. H.A.v.d.B is employed by a Plant Breeding company (Rijk Zwaan Breeding N.V.). The companies supporting this work have in no way influenced the results or conclusions drawn.

## Supplemental information

Data S1. Sample data repository, related to Step 69
