# Supplementary figures and images for: Protocol for capturing the RNA-binding proteome from plants using orthogonal organic phase separation
Source: STAR Protoc. 2025 Nov 27;6(4):104231. doi: 10.1016/j.xpro.2025.104231 (PMC12702337; doi:10.1016/j.xpro.2025.104231)

Unfiltered

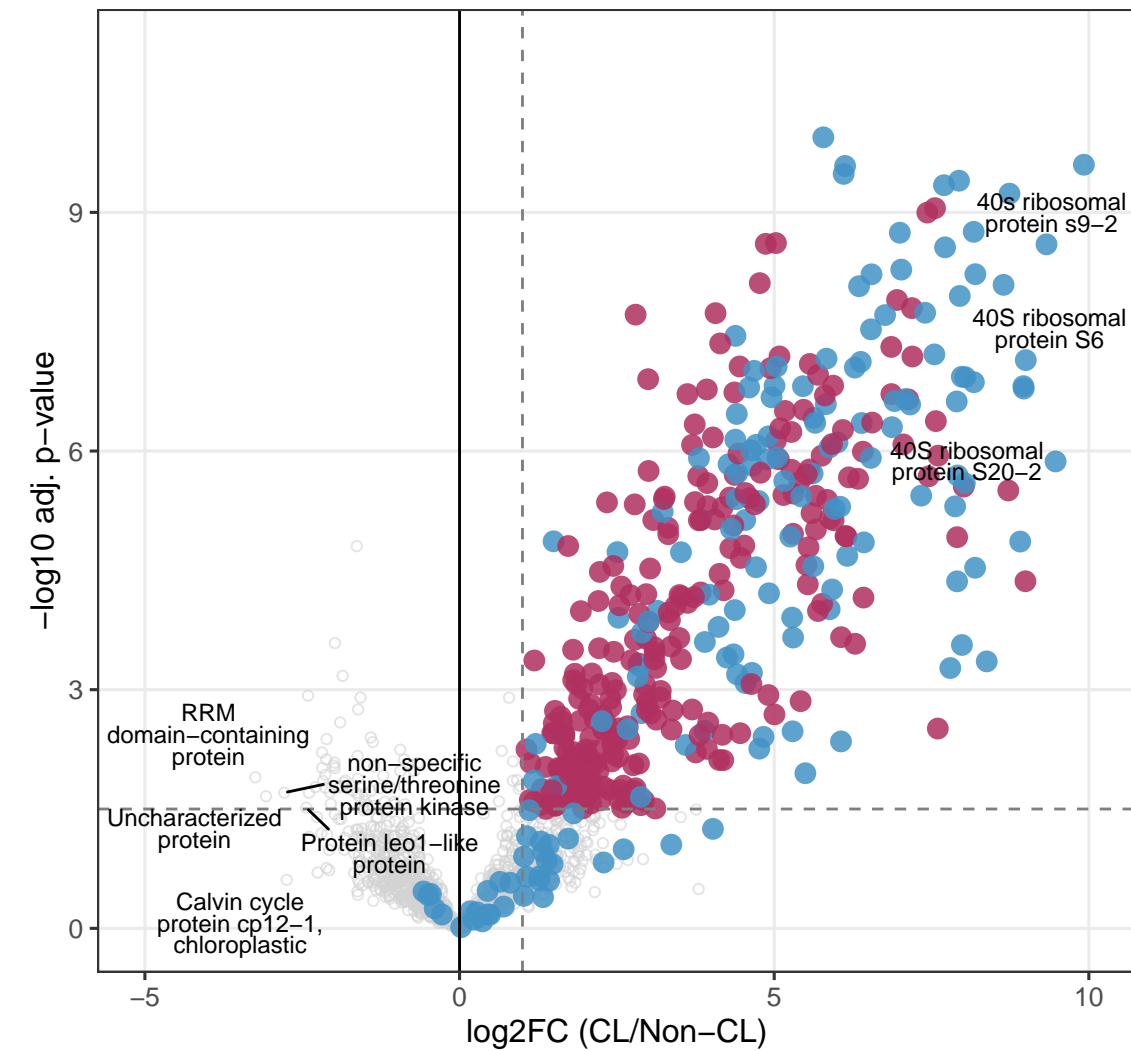

Filtered

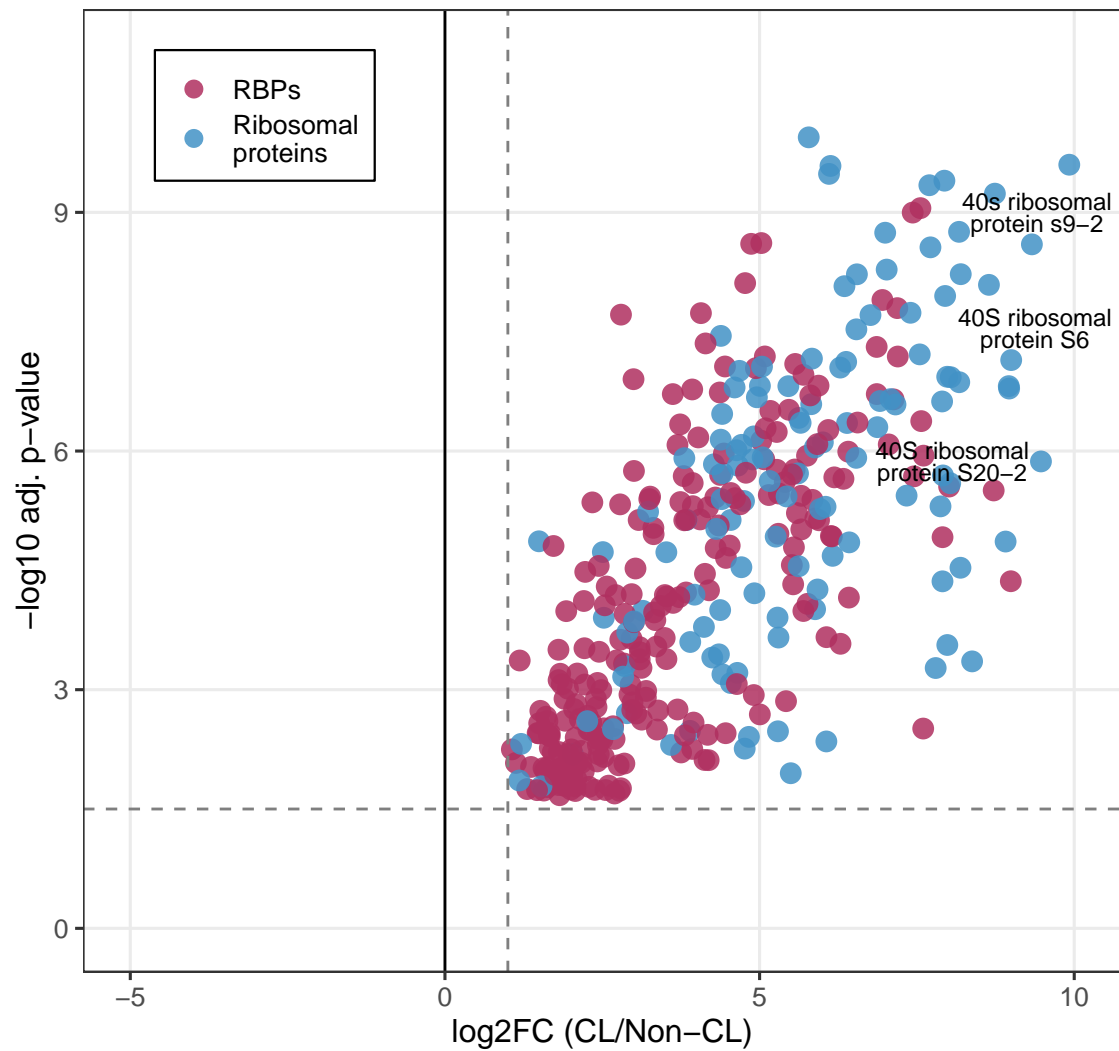

Supplement: Data S1. Sample data repository, related to Step 69 [file mmc1.zip › starprotocol_demo/expected_results/volcano_rbps_starprotocol.pdf]
